# Cognitive Vulnerabilities and Depression in Young Adults: An ROC Curves Analysis

**DOI:** 10.1155/2013/407602

**Published:** 2013-08-22

**Authors:** Michela Balsamo, Claudio Imperatori, Maria Rita Sergi, Martino Belvederi Murri, Massimo Continisio, Antonino Tamburello, Marco Innamorati, Aristide Saggino

**Affiliations:** ^1^DISPUTer, Dipartimento di Scienze Psicologiche, Umanistiche e del Territorio, “G. d'Annunzio” University, 66100 Chieti, Italy; ^2^Università Europea di Roma, 00163 Rome, Italy; ^3^Division of Psychiatry, Department of Neurosciences, University of Parma, 43100 Parma, Italy; ^4^Istituto Skinner, 00184 Rome, Italy

## Abstract

*Objectives and Methods.* The aim of the present study was to evaluate, by means of receiver operating characteristic (ROC) curves, whether cognitive vulnerabilities (CV), as measured by three well-known instruments (the Beck Hopelessness Scale, BHS; the Life Orientation Test-Revised, LOT-R; and the Attitudes Toward Self-Revised, ATS-R), independently discriminate between subjects with different severities of depression. Participants were 467 young adults (336 females and 131 males), recruited from the general population. The subjects were also administered the Beck Depression Inventory-II (BDI-II). *Results.* Four first-order (BHS Optimism/Low Standard; BHS Pessimism; Generalized Self-Criticism; and LOT Optimism) and two higher-order factors (Pessimism/Negative Attitudes Toward Self, Optimism) were extracted using Principal Axis Factoring analysis. Although all first-order and second-order factors were able to discriminate individuals with different depression severities, the Pessimism factor had the best performance in discriminating individuals with moderate to severe depression from those with lower depression severity. *Conclusion.* In the screening of young adults at risk of depression, clinicians have to pay particular attention to the expression of pessimism about the future.

## 1. Introduction

Major depressive disorder (MDD) is one of the most widespread psychiatric disorder [1–4] and the leading cause of disability as measured by years lived with disability (YLDs) [5–9]. Currently, MDD is estimated to be the fourth leading cause of global disease burden [10, 11], and, by the year 2020, it is projected to reach the second place in the ranking of the major causes of Disability Adjusted Life Years (DALYs). Today, depression is already the second cause of DALYs in the age category 15–44 years [11]. The estimated 12-month prevalence of MDD was 6.6% in the USA [12] and 3.0% in Italy [13]. 

Many approaches have been taken in attempts to explain the origins of depression [14]. Whereas some of these theories involved genetics and biological functioning, other approaches focused on the study of personal characteristics of individuals who are believed to be vulnerable to experiencing depressive episodes. A large body of research examining cognitive models of vulnerability for depression hypothesized that the way the individual interprets his/her experiences represents a protective or risk factor for the development of depressive disorders when negative stressful life events occur [15]. Depressed people engage in prolonged and repetitive thinking about the self, the world, and the future in a negative way with detrimental effects on mood [16–18]. Cognitive vulnerability (CV) for depression may be defined as a trait-like tendency to interpret information in a negative and distorted way when facing a subjectively perceived stressful event [19]. The literature indicated that CV may play a crucial role in the development and maintenance of depressive disorders not only in adulthood but also in childhood and adolescence (for a review see [20]). 

Despite similarities, currently there are several different theories which hypothesize the cognitive processes to be a diathesis for the development of depressive disorders. Two of the most renowned theories are the hopelessness theory [21] and Beck's theory [22, 23]. According to the hopelessness theory, three kinds of maladaptive inferences that people may make when confronted with negative events contribute to the development of hopelessness and, in turn, depressive symptoms: causal attributions, inferred consequences, and inferred characteristics about the self. Hopelessness and depressive symptoms are likely to occur when negative life events are (1) attributed to stable (i.e., likely to persist over time) and global (i.e., likely to affect many areas of life) causes; (2) viewed as likely to lead to further negative consequences; and (3) construed as implying that the person is unworthy or deficient. Similarly, Beck [22, 23] and Beck et al. [24] hypothesized depressogenic self-schemata, activated by the occurrence of negative life events, that take the form of overly pessimistic views of the self, the world, and the future (the *negative cognitive triad*). Also, Carver and Ganellen [25] considered self-punitiveness as a salient feature of depression, associated with the holding of overly high standards, the tendency to be too critical with the self for failing to attain a standard, and the tendency to generalize from a single failure to the broader sense of self-worth. To measure these three potential self-regulatory vulnerabilities to depression, Carver and colleagues developed the Attitudes Toward Self (ATS). 

Several researches have provided support for the critical role of CV in the origin and course of depressive disorders [26–30]. For example, Beckham et al. [26] reported that all the cognitive triad components are significantly associated with the severity of depressive symptoms in depressed patients. Evans et al. [28] indicated that holding a negative self-schema is an independent risk factor for the onset of depression in women. Furthermore, pessimistic views about the future have been found to be highly predictive of suicide behaviors [31, 32] and worse health and social functioning [33] in psychiatric patients. Again, in a longitudinal study, it was reported that undergraduate students with a more generalized negative cognitive style were 3.5–6.8 times more likely to report more depressive episodes compared to students with a less generalized negative cognitive style [29]. Some studies also indicated a partial overlap between the hopelessness and Beck's theories [34, 35].

Our literature review suggests that investigating the role of CV in the development of depression may be beneficial for a better understanding and treatment of depressive disorders. What is more is that research has failed to investigate the role of CV for depression in discriminating different levels of severity of depression. Thus, the aim of the present study was to evaluate, by means of ROC curves, whether some dimensions of CV for depression, as measured by three well-known instruments, that are the Beck Hopelessness Scale (BHS) [24], the Life Orientation Test-Revised (LOT-R) [36], and the Attitudes Toward Self-Revised (ATS-R) [37], discriminate independently between subjects with different severities of depression. 

## 2. Materials and Methods

### 2.1. Participants

Participants were 467 young adults (131 males, 28.1%; 336 females, 71.9%). Mean age of the sample was 23.58 ± 5.03 years (range 18–39). Subjects were included if they had an age between 18 and 40 years old. Exclusion criteria were the presence of any condition affecting the ability to complete the assessment, including illiteracy and denial of informed consent. 

All participants were nonrandomly recruited in Central Italy between February 2012 and June 2012. They accepted to participate voluntarily and gave their informed consent.

### 2.2. Measures

The Beck Depression Inventory-II (BDI-II) [38], the BHS, the LOT-R, and the ATS-R were administered to all subjects.

The BDI-II is a well-known self-report inventory composed of 21 items designed to assess the presence and severity of depressive symptoms, according to DSM-IV [39] criteria. Respondents endorse specific statements reflecting their feelings over the last two weeks, including today. Each statement is rated on a 4-point Likert-type scale ranging from 0 to 3, based on the severity of depressive symptoms. Importantly, extensive literature has supported the psychometric properties of the scale in clinical and nonclinical samples [40, 41]. In the current sample, Cronbach alpha was 0.86.

The BHS [24] is a 20-item scale for measuring the cognitive component of the depression. The scale assesses three major aspects of hopelessness: feelings about the future, loss of motivation, and expectations. Responding to the 20 true or false items, individuals have to either endorse a pessimistic statement or deny an optimistic statement. Research consistently have supported a positive relationship between BHS scores and measures of depression, suicidal intent, and current suicidal ideation [31, 42–44]. The Italian version of the BHS has reported good psychometric properties [45, 46].

The LOT-R is a 6-item scale measuring optimism: three items are positively worded (e.g., *“I'm always optimistic about my future”*), and the other three items are negatively worded (e.g., * “If something can go wrong for me, it will”*). Each item is rated on a five-point Likert-type scale (from 1—“I agree a lot”—to 5—“I disagree a lot”). Sum scores range between 6 and 30 with higher scores indicating higher optimism.

The ATS-R is a self-report measure designed to measure three potential self-regulatory vulnerabilities to depression. One of them is the holding of overly high standards, the second is the tendency to be self-critical at any failure to perform well, and the third is the tendency to generalize from a single failure to the broader sense of self-worth. The negative generalization scale has been found to be the dimension more strongly associated with depression [37, 47, 48]. Items are rated on a Likert-type scale, ranging from 1 (“*I agree a lot”*) to 5 (“*I disagree a lot”*). A study assessing the psychometric properties of the Italian version of the ATS-R has been recently published, indicating that the measure is a valid instrument for the study of the role of cognitive tendencies as potential diathesis in the development of depression [49].

### 2.3. Statistical Analysis

The BHS, LOT-R, and ATS-R items were subjected to Principal Axis Factoring in order to obtain common factors of vulnerability for depression. The number of latent factors to retain was selected using the scree test [50] and confirmed by means of Velicer's Minimum Average Partial (MAP) test [51]. Promax rotation with Kaiser Normalization was used in order to produce correlated factors. According to Kline [52], items were retained if they had a factor loading of 0.40 and higher and if they were loaded on a single factor. Then, factor scores for all subjects were calculated and used to assess the presence of possible higher-order factors. 

A generalized linear model with robust estimator was used to assess independent associations between depression severity and factors of vulnerability for depression. Associations were reported as odds ratio and their 95% confidence intervals (95% CI).

In order to assess the performance of the dimensions of vulnerability for depression in categorizing individuals on the basis of depression severity, a series of ROC test procedures were performed [53].

All analyses were performed with the Statistical Package for the Social Sciences (SPSS 17.0 for Windows).

## 3. Results

Descriptive statistics are listed in Table 1. The mean BDI-II score was 10.40 ± 8.36 (quartiles [25°/50°/75°]: 4/9/15), and 12.5% of the sample had scores of 20 and higher indicating moderate to severe depressive symptoms, consistent with the nonclinical nature of the sample.

The scree test and Velicer's MAP test indicated a factorial solution with four factors, explaining 47% of the variability of the data (Table 2). Nine items were loaded on the first factor (BHS Optimism), which explained 19.2% of the variance (eigenvalue = 6.92), with loadings ranging from 0.53 [BHS item no. 5] to 0.88 [BHS item no. 19]: 8 items from the BHS endorsing an optimistic statement and 1 item from the ATS-R denying higher standard for the self. On the second factor (BHS Pessimism), which explained 15.7% of the total variance (eigenvalue = 5.65), 9 items presented loadings ranging from 0.43 [BHS item no. 14] to 0.75 [BHS item no. 17]: all items were from the BHS and endorsed a pessimistic statement. On the third factor (Generalized Self-Criticism), which explained 7.3% of the variance (eigenvalue = 2.61), 4 items had loadings ranging from 0.62 [ATS-R item no. 9] to 0.75 [ATS-R item no. 10]: all items were extracted from the original Generalization and Self-Criticism factors of the ATS-R. On the fourth factor (LOT-R Optimism), which explained 4.8% of the variance (eigenvalue = 1.74), 3 items had loadings ranging from −0.51 [LOT-R item no. 10] to −0.69 [LOT-R item no. 4]: the items were from the LOT-R, and the individual had to endorse an optimistic statement. ATS-R items no. 3 and no. 6 loaded on two factors (factors no. 1 and no. 3) and were excluded from the solution. All factors were rated so that higher scores measured the presence of pessimism or negative attitudes toward the self or denied the presence of optimism. Correlation between factors ranged between −0.18, for the association between BHS Optimism and Generalized Self-Criticism, and 0.45, for the association between BHS Optimism and LOT-R Optimism. 

Factor scores were entered in a second-order factor analysis to extract higher-order common factors. The analysis resulted in two factors with eigenvalues >1. On the first factor (Optimism), which explained 43.3% of the variance (eigenvalue = 1.73), first-order factors LOT-R Optimism (0.80) and BHS Optimism (0.72) loaded. On the second factor (Pessimism/Negative attitudes toward self), which explained 34.0% of the variance (eigenvalue = 1.36), first-order factors BHS Pessimism (0.62) and Generalized Self-Criticism (0.69) loaded. The factors were weakly correlated with each other (*r* = 0.20).

A generalized linear model with robust estimator was performed to assess whether the factors measuring CV for depression were independently associated with the BDI-II (Table 3). The model fitted the data well (Likelihood Ratio *χ*_4_^2^ = 200.03; *P* < 0.001). The four factors were inserted as independent variables in the analysis and the BDI-II as criterion. Only the BHS Pessimism and the LOT-R Optimism were independently associated with BDI-II scores, indicating that (1) subjects with higher scores on the BHS Pessimism were 52.4 times more likely to have higher BDI-II scores (95% CI: 17.3/158.4; *P* < 0.001) compared with those reported having lower scores and (2) people with higher scores on the LOT-R Optimism were 9.1 times more likely to have higher BDI-II scores (95% CI: 3.9/21.6; *P* < 0.001) compared with people having higher scores. A second generalized linear model was performed with higher order factors as independent variables (not reported in the tables). The model fitted the data well (Likelihood Ratio *χ*_2_^2^ = 170.31; *P* < 0.001). Both the higher-order factors were independently associated with the BDI-II: (1) subjects with higher scores on Optimism were 10.1 times more likely to have higher BDI-II scores (95% CI: 4.7/21.5; beta = 2.31 [std. error = 0.39]; *P* < 0.001) compared with those having lower scores; and (2) people with higher scores on Pessimism/Negative Attitudes Toward Self were 91.1 times more likely to have higher BDI-II scores (95% CI: 32.7/254.3; beta = 4.51 [std. error = 0.52]; *P* < 0.001) than people reported having lower scores.

A series of ROC curves indicated that first-order and second-order factors of vulnerability for depression were able to discriminate individuals with different depression severities (Table 4). The performance of the BHS Optimism was quite stable at any level of depression. A randomly chosen individual had 57%–60% of probability to have higher scores on the BHS Optimism compared with a randomly chosen individual with lower levels of depression. Generalized Self-Criticism performance was the best when discriminating individuals with moderate to severe depression from other individuals: at these levels of depression, a randomly chosen individual with moderate to severe depression (BDI-II ≥ 20) had 67% of probability to have higher scores on Generalized Self-Criticism than a randomly chosen individual with lower levels of depression. The performance of the BHS Pessimism increased linearly from lower levels (BDI-II cutoffs of 5 and 9: AUC [area under the curve] = 0.72–0.74) to higher levels of depression (AUC of 0.81 and 0.87, resp., for BDI-II cutoffs of 15 and 19). The performance of the LOT-R Optimism was similar for lower cutoffs (AUC of 0.69 and 0.70, resp., for BDI-II cutoffs of 5 and 9) and higher cutoffs (AUC of 0.74 for BDI-II cutoffs of 15 and 19). When analyzing the performance of the second-order factors, a randomly chosen individual had 66%–72% of probability to have higher scores on Optimism than a randomly chosen individual with lower levels of depression, while a randomly chosen individual had 68%–81% of probability to have higher scores on Pessimism/Negative Attitudes Toward Self than a randomly chosen individual with lower levels of depression. Thus, the presence of Pessimism and Negative Attitudes Toward Self was more useful in discriminating individuals with moderate to severe depression than denying an optimistic one. However, the BHS Pessimism appeared to have the best performance in discriminating individuals at any level of depression severity.

## 4. Discussion

The aim of the present study was to evaluate, by means of ROC curves, whether CV for depression, as measured by three well-known instruments, may independently discriminate between subjects with different depression severities.

Our results indicated that the instruments we administered measure four common vulnerabilities for depression: (1) denying optimism/endorsing high standards (BHS/ATS-R); (2) endorsing pessimism (BHS); (3) generalizing self-criticism (ATS-R); and (4) denying optimism (LOT-R). These dimensions are loaded on two second-order factors: (1) denying optimism and (2) endorsing pessimism and generalizing self-criticism.

In our sample of young adults, the assessment of pessimism with the BHS, compared with other factors, had the best performance in discriminating individuals with different levels of depression severity, and its performance improved from lower cutoffs to higher cutoffs of depression. A randomly chosen individual with moderate to severe depression had 87% of probability to have higher BHS Pessimism compared with a randomly chosen individual with lower levels of depression. 

Pessimism was also independently associated with depression severity while controlling for other dimensions of vulnerability for depression. People having more severe pessimism were above 52 times more at risk to have higher depression compared with people with milder pessimism. This finding is consistent with the studies indicating a strong link between negative beliefs about the future and depression [54–58]. For example, Strunk et al. [56], investigating the relationship between depressive symptoms and bias in future event prediction, documented that individuals with elevated depressive symptoms showed a pessimistic bias in making predictions about the outcomes of future life events by overpredicting that undesirable events would happen to them and underpredicting that desirable events would happen to them. Alford et al. [59], in a sample of university students, found that in males hopelessness may predict specifically future depression severity but not anxiety. 

From a psychometric point of view, our results may be related to the differences in the methods employed in the three scales we administered to measure CV for depression: (1) the BHS uses dichotomous format of response, while for the LOT-R and the ATS-R the respondents are asked to rate each item on a Likert-type scale; (2) both the BHS and LOT-R ask the individuals to rate positive items which are directly associated with the construct they measure and negative statements which are negatively associated with these constructs. Furthermore, while the BHS is considered a measure of pessimism, the LOT-R is generally considered a measure of optimism [60]. 

Scheier and Carver [60], investigating construct validity of the LOT-R, reported the fit of a two-factor model: one factor contained positively worded items and the other included negatively worded items. The authors considered that the two-factor solution was probably due to item wording rather than content item [60]. Indeed, responses, particularly to the positively and negatively worded items, reflected a complicated combination of substantively meaningful trait effects and apparently idiosyncratic method effects associated with the wording of particular items [61–63].

Nevertheless, in the last decades several authors reported results supporting the hypothesis that Optimism and Pessimism are two distinct constructs with different patterns of correlations with other psychological constructs [64–70]. Recently, similar results have been found for the BHS: in a sample of medical patients, a bifactor model was the best-fitting solution and the most parsimonious among models evaluated [71]. Results from the higher-order factor analysis we performed support the hypothesis that the two factors often extracted from factorizing LOT-R and BHS items are not an artifact due to item wording and may have different roles as factors of vulnerability for depression. 

Finally, our study has some limitations. First, above 60% of our sample were composed of university students and may not be representative of the Italian population, so that generalizability of the results to older people and to individuals with low school attainment is not suggested. Second, our sample is nonclinical and only 12.5% of the subjects reported having moderate to severe depression at the BDI-II, so that our results may not be replicable in clinical samples, even though cognitive theories of depression did not predict differences in the associations of cognitive variables with depressive symptoms between clinical and nonclinical samples [21, 72, 73]. Third, the measures used in the present study were limited to only paper-and-pencil self-report instruments and we did not administered clinician-rated scales to measure depression, as recommended elsewhere [74]. Thus, our results may be biased by social desirability [75, 76]. Fourth, we did not assess participants for family history of mood disorders, which may have predisposed individuals to develop future depression and negative cognitive biases. Nevertheless, there are several noteworthy strengths, including the large size of sample and the use of three different measures often used to assess cognitive vulnerability for depression.

In conclusion, when screening young adults at risk for depression, clinicians have to pay particular attention to expressions of pessimism about the future, which may discriminate well individuals with moderate to severe depression from individuals reported having lower depression severity. Furthermore, at these levels of depression severity, Generalized Self-Criticism is less effective in discriminating individuals at risk for depression compared not only with statements of pessimism but also with statements denying optimism. 

## Figures and Tables

**Table 1:** Characteristics of the sample.

	%
Men	28.2
Age—M ± SD	23.59 ± 5.03
Job	
Employed	32.9
Students	63.7
Unemployed	3.4
Marital status	
Not married	87.8
Married	10.8
Divorced	1.4
Education	
5 years	0.6
8 years	12.3
13 years	72.0
16+ years	15.1
BDI-II—M ± SD	10.40 ± 8.36
BDI ≥ 20	12.5

**Table 2:** Factor solution (Principal Factor Analysis, Promax rotation with Kaiser Normalization).

	Factor			
	BHS Optimism	BHS Pessimism	Generalized Self-Criticism	LOT Optimism
BHS no. 1	0.67	—	—	—
BHS no. 2	—	0.53	—	—
BHS no. 3	0.75	—	—	—
BHS no. 4	—	—	—	—
BHS no. 5	0.53	—	—	—
BHS no. 6	0.82	—	—	—
BHS no. 7	—	0.69	—	—
BHS no. 8	—	—	—	—
BHS no. 9	—	0.61	—	—
BHS no. 10	0.66	—	—	—
BHS no. 11	—	0.68	—	—
BHS no. 12	—	0.49	—	—
BHS no. 13	0.70	—	—	—
BHS no. 14	—	0.43	—	—
BHS no. 15	0.55		—	—
BHS no. 16	—	0.74	—	—
BHS no. 17	—	0.75	—	—
BHS no. 18	—	—	—	—
BHS no. 19	0.88		—	—
BHS no. 20	—	0.70	—	—
ATS no. 1	−0.74	—	—	—
ATS no. 2	—	—	0.69	—
ATS no. 3	−0.55	—	0.47	—
ATS no. 4	—	—	—	—
ATS no. 5	—	—	—	—
ATS no. 6	−0.50	—	0.53	—
ATS no. 7	—	—	—	—
ATS no. 8	—	—	0.66	—
ATS no. 9	—	—	0.62	—
ATS no. 10	—	—	0.75	—
LOT no. 1	—	—	—	−0.62
LOT no. 3	—	—	—	—
LOT no. 4	—	—	—	−0.69
LOT no. 7	—	—	—	—
LOT no. 9	—	—	—	—
LOT no. 10	—	—	—	−0.51

Cronbach alpha	0.84	0.84	0.78	0.70

Extraction method: Principal Axis Factoring. Rotation method: Promax with Kaiser Normalization.

**Table 3:** Generalized linear model (criterion: BDI-II).

Parameter	Beta	Std. error	OR	95% Wald confidence interval for OR		*P*<
				Lower	Upper	
BHS Optimism	0.12	0.36	1.13	0.55	2.31	0.74
BHS Pessimism	3.96	0.56	52.35	17.31	158.37	**<0.001**
Generalized Self-Criticism	0.33	0.47	1.39	0.56	3.47	0.48
LOT Optimism	2.21	0.44	9.13	3.87	21.56	**<0.001**

Likelihood Ratio *χ* ^2^_4_ = 200.03; *P* < 0.001; AIC = 3013.74; AICC = 3013.93; Pearson *χ* ^2^_448_ = 20017.73; value/DF = 44.68.

**Table 4:** ROC curves.

Variables	Area under the curve	Std. error	Asymptotic sig.
BDI-II > 5			
BHS Optimism	0.57	0.03	0.05
BHS Pessimism	0.72	0.03	<0.001
Generalized Self-Criticism	0.58	0.03	0.01
LOT Optimism	0.69	0.03	<0.001
Optimism	0.66	0.03	<0.001
Pessimism/Negative Attitudes Toward Self	0.68	0.03	<0.001
BDI-II > 9			
BHS Optimism	0.58	0.03	0.01
BHS Pessimism	0.74	0.02	<0.001
Generalized Self-Criticism	0.59	0.03	0.001
LOT Optimism	0.70	0.02	<0.001
Optimism	0.67	0.03	<0.001
Pessimism/Negative Attitudes Toward Self	0.70	0.03	<0.001
BDI-II > 15			
BHS Optimism	0.60	0.03	0.01
BHS Pessimism	0.81	0.02	<0.001
Generalized Self-Criticism	0.60	0.04	0.01
LOT Optimism	0.74	0.03	<0.001
Optimism	0.72	0.03	<0.001
Pessimism/Negative Attitudes Toward Self	0.75	0.03	<0.001
BDI-II > 19			
BHS Optimism	0.59	0.03	0.05
BHS Pessimism	0.87	0.02	<0.001
Generalized Self-Criticism	0.67	0.04	<0.001
LOT Optimism	0.74	0.03	<0.001
Optimism	0.71	0.03	<0.001
Pessimism/Negative Attitudes Toward Self	0.81	0.03	<0.001
